# Prevalence of Undiagnosed Diabetes and Prediabetes in the Dental Setting: A Systematic Review and Meta-Analysis

**DOI:** 10.1155/2020/2964020

**Published:** 2020-08-25

**Authors:** Alagesan Chinnasamy, Marjory Moodie

**Affiliations:** ^1^Melbourne Dental School, The University of Melbourne, Melbourne 3053, Australia; ^2^Deakin Health Economics, Deakin University, Waurn Ponds, Geelong, Australia

## Abstract

**Background:**

With the close link between diabetes mellitus (DM) and periodontal disease (PD), dentists have an unrealized opportunity to make a chance discovery of a patient's medical condition. Unlike in the medical setting, information on the point of care (PoC) and opportunistic screening for DM in the dental setting is limited. To make a reliable estimate on the prevalence of undiagnosed type 2 diabetes mellitus (T2DM) and prediabetes among dental patients in the dental setting and to assist healthcare planners in making an informed decision, information on the disease frequency and strategies employed to address this issue is of paramount importance.

**Objectives:**

To summarize the data on the prevalence of undiagnosed T2DM and prediabetes amongst dental patients and further explore the effectiveness of the PoC screening and its implication for use in the dental setting.

**Methods:**

A MEDLINE-PubMed, EMBASE, Web of Science, and Cochrane Library search was conducted with no time specification. Information on study characteristics and diagnostic parameters was retrieved for meta-analysis. All the studies were assessed for methodological quality using the QUADAS-2 tool. Proportions were presented in tables and forest plots. All statistical analysis was performed using the MedCalc software.

**Results:**

Nine studies met the inclusion criteria. The proportion of dental patients identified to be at a risk of hyperglycaemia with the PoC screening using random blood glucose (RBG) and HbA1 was 32.47% and 40.10%, whilst the estimated proportion with undiagnosed T2DM and prediabetes was identified as 11.23% and 47.38%.

**Conclusion:**

A significant proportion of dental patients can be identified as undiagnosed T2DM and prediabetes. Targeted opportunistic screening is a feasible approach and can help reduce the prevalence of undiagnosed T2DM and prediabetes.

## 1. Introduction

The World Health Organization (WHO) estimates hyperglycaemia to be the third highest risk factor for premature mortality [1–4]. Type 2 diabetes mellitus (T2DM) is the most prevalent form of diabetes mellitus (DM) and accounts for almost all cases of undiagnosed DM. Globally, 463 million (9.3% of the global population) are affected by DM. Half of those (232 million) were unaware of their disease status [2, 5, 6].

People at the risk of developing DM are in a lengthy asymptomatic phase that precedes overt DM [3, 7]. Combination of factors, including slow onset of symptoms, underperforming health care system, low awareness among people, and varied presentation of symptoms, often made the physician and the patient difficult to determine that overlooked DM as the cause, leading to late diagnosis [4, 5, 8–10].

Hyperglycaemia affects nearly every organ in the body, and if left uncontrolled, complications become established in the continuum from prediabetes to DM. With no known cure, the starting point of living well with DM is early diagnosis. Hence, screening is the first stage in the care continuum [5].

Centralised laboratory testing does not represent a convenient process for the patient, often requiring more than one visit. Patient-centred healthcare is becoming a global trend and is based on the foundation that health care should be organized closer to the consumer rather than the providers [11–13]. Point of care (PoC) denotes testing done close to the patients. It offers opportunity for early diagnosis and prevention that may reduce the number of late diagnoses, costs of hospitalization, and filter access to specialist care and monitoring of DM [3, 14]. In the Western world, a 66% reduction in the proportion of undiagnosed DM [15, 16] is attributed to opportunistic screening in primary care [17, 18]. The United Kingdom National Screening Committee guidelines recommends that opportunistic screening is cost-effective and valuable only when it is used in high-risk individuals or groups [4, 9, 10, 19, 20]. The identification of patients with undiagnosed T2DM [21] in the dental setting represents one such group.

Periodontal disease (PD) is one of the leading cause of tooth loss in adults, and DM is an established risk factor for PD. Dental patients with poorly controlled DM experience far greater periodontal problems and poorer treatment outcomes compared to those who maintain their blood glucose within normal limits [2, 12, 22, 23]. With this close link between DM and PD, dentists have an unrealized opportunity to make a chance discovery of patients' medical problems, identify risk groups, and refer them to physicians for further evaluation and care.

Unlike in the medical setting, information on PoC opportunistic screening for DM in the dental setting is limited, and no previous study had reported a proportion of undiagnosed T2DM in the form of meta-analysis. To make a reliable estimate on the risk of DM among dental patients and to assist healthcare planners in making informed decisions, information on the disease prevalence and strategies to address this issue is of paramount importance. As such, the aim of the current study is to summarize the data on the prevalence of undiagnosed T2DM and prediabetes among dental patients and explore PoC screening and its implication for use in the dental setting.

## 2. Methods

We followed the Preferred Reporting Items for Systematic Reviews and Meta-Analyses (PRISMA) guideline for conducting this study.

### 2.1. Search Strategy and Study Selection

A search strategy was developed using the population, intervention, comparison, and outcome (PICO) approach to address the review question “What is the diagnostic yield of PoC screening for T2DM in the dental setting?”

A MEDLINE-PubMed, EMBASE, Web of Science, and Cochrane Library search, limited to the English language, was performed with no time specification. A comprehensive search strategy, including qualified Medical Subject Headings (MeSH Terms) and keywords or free text words in simple or multiple conjunctions using the Boolean operator (AND/OR), was conducted. Search terms were grouped into categories for “Problem,” “Intervention,” and “Outcome.”

### 2.2. MEDLINE-PubMed Search Strategy

(((((((((((adult onset diabetes mellitus[MeSH Terms]) OR hyperglycemia[MeSH Terms]) OR prediabetes[MeSH Terms]) OR diabetic) OR undiagnosed) AND Humans[MeSH] AND adult[MeSH])) NOT gestational diabetes[MeSH Terms]) AND Humans[MeSH] AND adult[MeSH])) AND ((((dental auxiliaries[MeSH Terms]) OR (dentist^*∗*^ or dental)) OR periodont^*∗*^) OR “oral health professional^*∗*^”)) AND (((((screen^*∗*^) OR hba1[MeSH Terms]) OR blood glucose[MeSH Terms]) OR identif^*∗*^[MeSH Terms]) OR point of care)

### 2.3. OVID-EMBASE Search Strategy

1. (Diabetes or prediabetes or hyperglycemia^*∗*^)
2. (screen^*∗*^ or examin^*∗*^ or identif^*∗*^ or “point of care”)
3. (adult^*∗*^ or patient^*∗*^ or individuals^*∗*^ or people^*∗*^)
4. (dentist^*∗*^ or dental or “oral health professional^*∗*^”)

### 2.4. Outcome Measures

The proportion of adult dental patients with undiagnosed T2DM or prediabetes (hyperglycaemia) was estimated.

### 2.5. Study and Participant Selection

Studies were eligible for inclusion if

- They were cross-sectional in design and used PoC screening for undiagnosed DM or hyperglycaemia in the dental setting. This includes studies that used blood glucose investigation and glycated haemoglobin (HbA1c).
- The results of the PoC screening test were confirmed with the reference test (diagnostic test). The reference test includes all currently available criteria and methods recommended by the WHO or the American Diabetes Association (ADA) for the diagnosis of DM or hyperglycaemia that is not suggestive of DM.

Studies were excluded if

- They used PoC screening based on reagent strips that rely on colour coding.
- They relied on PoC HbA1c for diagnosis, as the current WHO and the ADA guidelines do not recommend point-of-care HbA1c for diagnostic purposes [24, 25].

### 2.6. Participants' Inclusion Criteria

Adult patients accessing dental care for routine dental treatment, who had never been told by a healthcare provider that they have DM or hyperglycaemia, were included.

### 2.7. Participants' Exclusion Criteria

- Pregnant women
- Patient with previous diagnosis of DM or prediabetes
- Unable to provide consent
- Severe physical or mental impairment
- Serious heart or renal disorder
- Systemic steroidal use

### 2.8. Data Extraction and Quality Assessment

Two reviewers extracted information from selected papers on author, country, year, number of participants with undiagnosed T2DM or hyperglycaemia, age, eligibility criteria, exclusion criteria, gender distribution, type of PoC screening, and diagnostic test and the threshold used. Any discrepancy or missing information was discussed, and a consensus was reached between the authors. Studies that met the criteria (Table 1) were used to categorize the patients into one of the three categories: (a) normoglycemia, (b) prediabetes, and (c) DM [24, 25]. The screening test includes all PoC devices for measuring blood glucose or HbA1c, and the diagnostic tests include laboratory HbA1c, oral glucose tolerance test (OGTT), and fasting plasma glucose (FPG).

A systematic review [26] concluded with no consensus reached on the tools to assess the quality of the observational study methodology investigating prevalence. Further, the Cochrane collaboration recommends a domain-based evaluation to assess the risk of bias on a subjective basis [27]. Hence, we chose the Quality Assessment of Diagnostic Accuracy Studies (QUADAS-2) tool [28] for observational studies to perform the quality assessment. QUADAS-2 comprises four domains: patient selection, index test, reference standard, and flow of patients and timing. Individual articles were assessed against each domain to identify the risk of bias.

### 2.9. Data Synthesis and Analysis

Meta-analysis was performed to calculate the weighted prevalence and presented in forest plots or tables with 95% confidence intervals (CI). The screening test used blood glucose investigation and glycated haemoglobin that differed significantly in their threshold. To avoid variation in the PoC screening, a subgroup analysis was conducted for the RBG and HbA1c tests. For the diagnostic test, no subgroup analysis was done since the diagnosis is based on the criteria adopted by the WHO and the ADA.

The PoC test results were grouped into low (normoglycemic range) and high-risk (hyperglycaemic range). The diagnostic test results were categorized into prediabetes and T2DM based on the reported values. To identify the proportion of dental patients with undiagnosed T2DM or hyperglycaemia, a Freeman–Tukey transformation, arcsine square root transformation [29], was used to calculate the weighted summary proportion under the random-effect model [30]. Heterogeneity among studies was reported using the *I*^2^ statistic. All analyses were performed using MedCalc 12.1.4.0 statistical software (MedCalc Software, Mariakerke, Belgium).

## 3. Results

**Figure 1:**
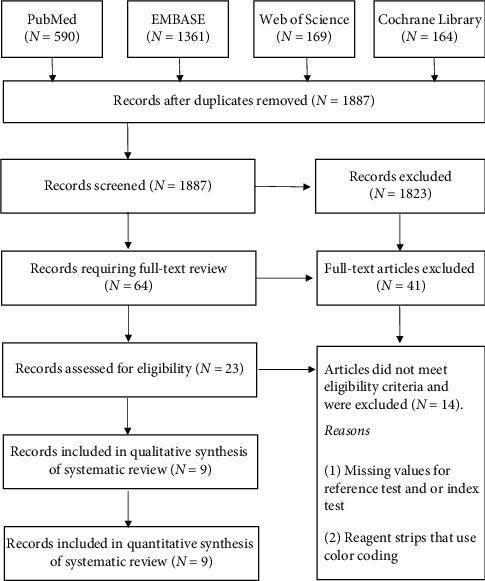
Study selection flow diagram.

The flowchart in Figure 1 summarises the selection process. The search identified 1887 articles after the removal of duplicates; title and abstract search excluded 1823 records due to nonrelevance, 64 were selected for full-text review, and 41 were excluded as they did not meet the study requirements. Of the 23 records assessed for eligibility, 12 [2–4, 9, 10, 12, 13, 31–35] did not report the diagnostic test results and another two [6, 36] used PoC screening with reagent strips that use colour coding to identify the risk of T2DM. The remaining nine studies were included in the analysis.

### 3.1. Methodological Quality of Included Studies

**Figure 2:**
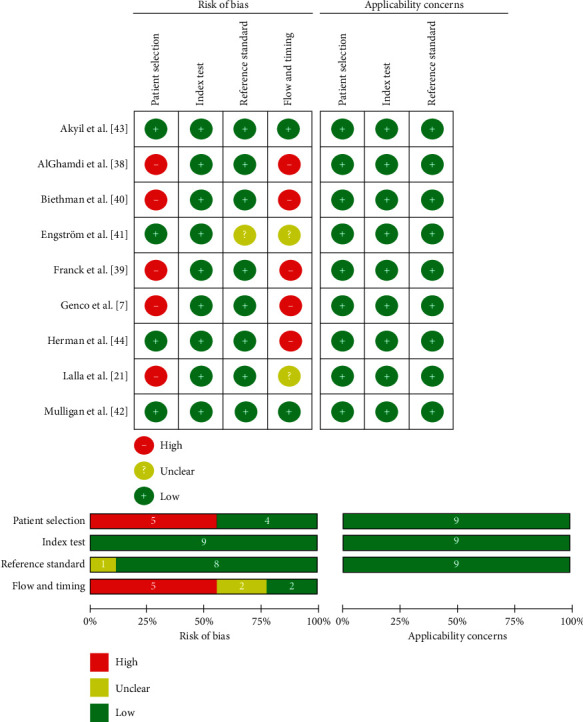
Summary of risk of bias and applicability concerns. Review of authors' judgment.

Review Manager 5.3 tool from the Cochrane collaboration was used to prepare the figures for the risk of bias and applicability concern (QUADAS-2). Results of the methodological quality are presented in Figure 2. Five [7, 21, 37–39] of the nine studies were identified as involving a significant risk of bias with the patient selection process; none of them used consecutive or random sampling in patient selection. One study [38] used a predefined age range 44 to 57 years and waist-hip measurement for inclusion. Only one study [37] of the nine mentioned blinding (double), but still scored high on the risk of bias in patient selection process. Two studies included all the participants for the screening and diagnostic test; one of these studies [40] did not report the name of the diagnostic test done. None of the included studies was identified as an applicability concern in all the three domains of the QUADAS-2 tool.

### 3.2. Characteristic of Included Studies

Summary characteristics of the included studies are presented in Table 2. Nine studies met the inclusion criteria. All were cross-sectional observational in design. Six were from the United States and one each from Sweden, Saudi Arabia, and Turkey. The age range of the participants was 20 to 75 years. For the screening test, HbA1c was used in four of the studies and the remaining used RBG. Eight [7, 21, 37, 39–43] of the nine studies used a risk assessment tool or questionnaire in some capacity, but only one [38] used it as a prerequisite for risk stratification to be eligible for PoC screening. One study [21] did not report on the number of screen positives with the PoC screening.

### 3.3. PoC Screening Test Outcome

**Figure 3:**
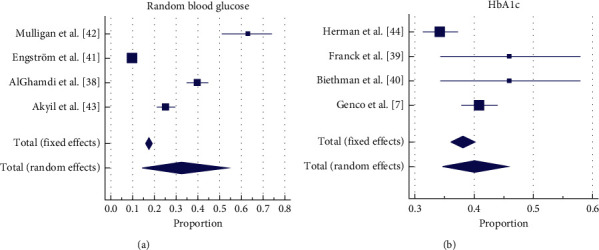
Proportion of screen positives (above the normoglycemic range) in dental patients.

Eight of the nine studies were included in the analysis. The weighted proportions of patients screened positive for undiagnosed T2DM or hyperglycaemia are summarised in Figure 3. The number of dental patients screened with the RBG and HbA1c was 2,462 and 2,198, respectively. Two of the included studies were comparative [41, 42], using different screening strategies on the same patient. The weighted summary proportion under the random-effect model for the RBG was 32.47% (95% CI = 14.32% to 53.90%) and 40.10 (95% CI = 34.62% to 45.72%) for HbA1c. Heterogeneity for the RBG was *I*^2^ = 98.85% (CI = 98.23 to 99.25) and HbA1c was *I*^2^ = 78.18 (CI for *I*^2^ = 41.14 to 91.91), respectively.

### 3.4. Diagnostic Test Outcome

**Figure 4:**
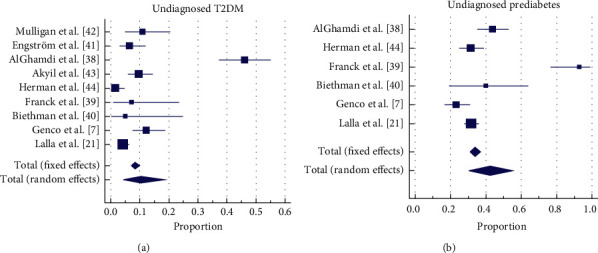
Undiagnosed T2DM and prediabetes.

All the dental patients who had the diagnostic test from the nine studies (*N* = 1429) were included in the meta-analysis for undiagnosed T2DM. The weighted proportion (Figure 4) confirmed with the reference standard is 10.40% (95% CI = 4.38% to 18.60%) and a heterogeneity statistic of *I*^2^ = 94.41%; (95% CI = 91.38% to 96.38%). Only six of the nine studies reported hyperglycaemia not suggestive of T2DM, and the weighted proportion (Figure 4) was 42.45% (30.26% to 55.12%) with a heterogeneity statistic of *I*^2^ = 92.24% (95% CI = 85.86% to 95.74%).

Based on the reported value, the estimates for undiagnosed T2DM and hyperglycaemia (Table 3) in the dental setting were calculated, assuming that all the screen-positive dental patients (*N* = 1,302) presented, with no loss of follow-up, for the diagnostic reference standard. For undiagnosed T2DM, eight of the nine studies were included, and for prediabetes, four of the nine studies were included in the analysis. With this assumption, the estimated proportion of undiagnosed T2DM and hyperglycaemia with the PoC was 11.23% (95% CI 3.93% to 21.63%) and 47.38 (95% CI 27.25% to 67.97%). Only the screen-positive PoC HbA1c was included when estimating hyperglycaemia. This was done because only two of the four studies that used PoC RBG reported hyperglycaemia that was confirmed with the reference standard. Further, to avoid any inconsistency in the estimate of prediabetes with two different screening tests, the PoC RBG test results were excluded in this analysis.

## 4. Discussion

Globally, 9.3% of adults aged 20 to 79 years are estimated to have DM, inclusive of undiagnosed DM [5]. With 50% of the DM being undiagnosed [5], our estimate of 11.23% in the dental setting is more than twice the global prevalence of undiagnosed DM. Our findings are essentially based on studies from four countries, of which approximately 55% of the samples (*N* = 2806) were from the United States (US); except for one study that did not report on the screen positives, the proportion of dental patients at risk of hyperglycaemia (38.9%, *N* = 884) is mainly in line with the prevalence of undiagnosed DM (38.2%) in the US [44].

Several studies have shown that health professionals from different specialities have contributed to the identification of DM [37, 45, 46]. The findings suggest that a significant proportion of the dental patients can be identified as at a risk of hyperglycaemia with PoC opportunistic screening. Most people visit their dentists when they perceive themselves as not unhealthy but visit the physician while they are sick [47]. A recent systematic review update on the bidirectional relationship between PD and T2DM concluded a higher risk of incident T2DM among those with periodontitis compared to periodontally healthy individuals [48]. This gives oral health professionals (OHP) an opportunity to identify asymptomatic patients with underlying medical conditions. Routine oral health screenings can be extended to systematically screen for a particular disease such as DM. By stretching the number of contact points between the health care providers and individuals seeking care, there is plenty of opportunity for early detection of asymptomatic individuals at risk of DM with PoC screening. Shared responsibility in early identification of disease will also lessen some of the load imposed on the medical community.

**Figure 5:**
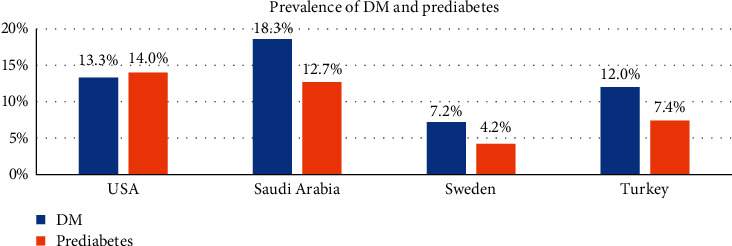
Prevalence of T2DM and IGT for selected countries.

The nine studies analysed come from three high-income countries (Sweden, US, and Saudi Arabia) and one upper-middle-income (Turkey) country [49]. Their combined national prevalence for DM and IGT is 12.7% and 9.6%, respectively (Figure 5) [5, 50]. The International Diabetes Federation (IDF) estimates 38.3% of all the DM as undiagnosed in the high-income countries [5]. This would translate into a pooled national prevalence of undiagnosed DM as 4.9% for the selected countries. Our findings' estimated prevalence is 2.3 and 4.9 times more than the prevalence of undiagnosed DM and hyperglycaemia for the selected countries.

Prediabetes is an asymptomatic precursor for DM and is gaining increasing attention because the progression from prediabetes to T2DM is not inevitable. The average duration of prediabetes among individuals 30 years and older is 8.5 years in males and 10.3 years in females [51]. Early identification of this condition provides a window of opportunity to alter the natural history of T2DM. Several DM prevention programs have identified lifestyle interventions that potentially provide sustained benefits for many years, with a lower rate of progression from prediabetes to T2DM [52]. Our estimate identified that 47.3% of the screen-positive dental patients were identified as prediabetic with the reference standard. It is therefore vital that prediabetes is identified early, particularly in the presence of mild hyperglycaemia, so that early intervention can prevent or delay the onset of DM with minimal micro- and macrovascular complications [53, 54].

Strauss and colleagues identified that more than 90% of the patients with periodontal disease met the ADA guidelines for diabetes screening; of those, around 60% had seen a dentist in the last two years [55]. Risk stratification using the noninvasive T2DM risk assessment questionnaire followed by a PoC screening increases the proportion of positive findings. With the two-step screening approach, the number needed to screen to identify true positive cases will be much lower and cost-effective [20, 56]. However, patient compliance for physician referral was identified as a significant concern in a study conducted in the US with only 21.5% of the dental patients from private dental clinics following up with their physician despite an HbA1c value of ≥5.7 and being informed prior of the possibility of being referred to the physician. The reasons for nonadherence are not clear, but resistance to compliance is a significant barrier, and strategies to overcome this need to be explored. Genco et al. recommended either a formal contract with the patient to follow-up with a referral or the involvement of more OHP to monitor the entire process including referral and follow-up [7]. Such concerns need to be explored and addressed to establish and streamline this extended service in the dental setting. To implement T2DM screening in the dental setting, it is important for OHP to appreciate the value, willingness to screen, and patient compliance.

### 4.1. Strength and Limitations

To our knowledge, this is the first meta-analysis on the prevalence of undiagnosed T2DM and hyperglycaemia in the dental setting. Nevertheless, it has some limitation. We may have missed a few relevant studies by restricting to published literature from the core electronic databases. Observational studies are prone to high heterogeneity [57]. The heterogeneity in our study is primarily due to variation in the demographic distribution of the study samples. In addition, differing sample sizes with diverse disease prevalence is an important limitation in making an accurate estimate of the prevalence of undiagnosed diabetes in the dental setting. However, to minimize sampling and threshold variation between studies and make the results more informative, subgroup meta-analysis for the PoC screening was performed. Two of the studies [37, 38] acted as a potential source of heterogeneity; the study done in Saudi Arabia identified nearly 40% of all those screened with the index test as having undiagnosed T2DM. The author mentioned the limitation that a large proportion of the samples were from the lower socioincome group. As Saudi Arabia accounts for the second highest prevalence of DM in the Middle East and North African (NEMA) region [5], the findings seem to truly reflect the disease burden. The other study [38] had a small sample size and used a two-stage risk stratification before the patient took the diagnostic test. As such, we did not find any reason to exclude these two studies from the analysis.

Countries with high prevalence of DM also account for the higher number of undiagnosed DM. This is of particular relevance to our findings as six of the nine studies are from the US, where prevalence of DM and IGT is among the highest in the world. Because of this disparity, a blanket extrapolation to generalize the prevalence of undiagnosed T2DM in the dental setting needs to be interpreted with caution.

One study [41] included all the samples for the diagnostic test follow-up despite the screen test outcome. To avoid overestimation and reflect the true potential of PoC screening and its limitation in the missed diagnosis, only those patients who were screened positive were selected to estimate the disease outcome. Despite this study being carried out in the 1990, the reference standard used (OGTT) is a time tested method for the diagnosis of DM. As such, we did not see that as a threat that would undermine the findings.

## 5. Conclusion

The estimated prevalence of undiagnosed T2DM and prediabetes in the dental setting is 11.23% and 47.38%. Targeted PoC screening in the dental setting is a novel approach that can potentially help in reducing the prevalence of undiagnosed hyperglycaemia, thereby leading to early referral to the physician, reduced complications with earlier treatment, improved quality of life, improved productivity, and cost saving for both individuals and the government. OHP can also play an essential role in creating awareness about DM and promoting healthy lifestyles and habits by enhancing patients' understanding of the potential oral health consequences associated with DM. More research is needed to augment on the expanded scope for PoC screening for T2DM in the dental setting to demonstrate utility and effectiveness particularly in the low- and middle-income countries where the prevalence of undiagnosed T2DM is high.

## Figures and Tables

**Table 1:** Diagnostic criteria for DM and hyperglycaemia adopted by the WHO and ADA.

**Diabetes**	
FPG	≥7.0 mmol/L (126 mg/dl) or
2-hour OGTT	≥11.1 mmol/L (200 mg/dl) or
HbA1c	≥6.5%

**Hyperglycaemia (prediabetes range)**	
*Impaired glucose tolerance (IGT)*	
FPG	<7.0 mmol/L (126 mg/dl)
2-hour OGTT	≥7.8 and <11.1 mmol/L (140 mg/dl and 200 mg/dl)
*Impaired fasting glucose (IFG)*	
Fasting plasma glucose	6.1 to 6.9 mmol/L (110 mg/dl to 125 mg/dl)
2-hour OGTT	<7.8 mmol/L (140 mg/dl)
*HbA1c*^*∗*^	A1C ≥ 5.7 to 6.4% (39–47 mmol/mol)

^*∗*^Criteria recommended by the American Diabetes Association alone; FPG, fasting plasma glucose; OGTT, oral glucose tolerance test; HbA1c, glycated haemoglobin; IFG, impaired fasting glucose; IGT, impaired glucose tolerance.

**Table 2:** Characteristics of the included studies.

Lead author	Country	Patient age range	Mean age	Screening strategy	Index test (PoC)		Reference test		
					Number screened	Screen positive	Number attended	Diagnosed	
								PD	DM
Mulligan et al. [41]	USA	≥65	74.2	RBG (PoC) > OGTT	73	46	73	—	8
Lalla et al. [21]	USA	≥30	53	HbA1c (PoC) > FPG	535	n/a	506	161	21
Engström et al. [40]	Sweden	20–75	48.6	RBG (PoC) > n/a	1568	155	139	—	9
AlGhamdi et al. [37]	SA	≥40	48.9	RBG (PoC) > OGTT	385	153	128	56	59
Akyil et al. [42]	Turkey	≥20	n/a	RBG (PoC) > OGTT	436	88	208	—	20
Genco et al. [7]	USA	≥45	55	HbA1c (PoC) > HbA1c	1017	416	146	34	18
Franck et al. [38]	USA	n/a	n/a	HbA1c (PoC) > HbA1c	74	34	28	26	2
Herman et al. [43]	USA	≥30	52.8	RBG (PoC) > HbA1c	1033	354	181	57	3
Biethman et al. [39]	USA	≥30	59.6	HbA1c (PoC) > n/a	74	34	20	8	1

RBG, random blood glucose; FPG, fasting plasma glucose, PoC, point of care; HbA1c, glycated haemoglobin; OGTT, oral glucose tolerance test; PD, prediabetes; DM, diabetes mellitus.

**Table 3:** Estimated undiagnosed T2DM and hyperglycaemia in the dental setting.

Included studies	Sample size	Proportion (%)	95% CI	Weight (%)
				Random
*Prediabetes estimate*				
Herman et al. [43]	354	31.35	26.55 to 36.47	26.73
Franck et al. [38]	34	94.11	80.32 to 99.28	23.24
Biethman et al. [39]	34	41.17	24.64 to 59.30	23.24
Genco et al. [7]	416	23.31	19.33 to 27.68	26.79
Total (random effects)	838	47.38	27.25 to 67.97	100.00

*Undiagnosed T2DM estimate*				
Mulligan et al. [41]	46	10.87	3.62 to 23.57	11.88
Engström et al. [40]	155	6.452	3.13 to 11.54	12.98
AlGhamdi et al. [37]	153	46.40	38.31 to 54.63	12.97
Akyil et al. [42]	110	10.00	5.09 to 17.18	12.77
Herman et al. [43]	354	1.695	0.62 to 3.65	13.27
Franck et al. [38]	34	5.882	0.72 to 19.67	11.41
Biethman et al. [39]	34	5.882	0.72 to 19.67	11.41
Genco et al. [7]	416	12.26	9.26 to 15.80	13.31
Total (random effects)	1302	11.23	3.93 to 21.63	100.00

## Data Availability

The datasets generated and/or analysed during the current study are publicly available from the included studies. Data can also be provided from the corresponding author on reasonable request.
